# Supplementary material for: Validation of UHPLC-ESI-MS/MS Method for Determining Steviol Glycoside and Its Derivatives in Foods and Beverages
Source: Foods. 2023 Oct 27;12(21):3941. doi: 10.3390/foods12213941 (PMC10647612; doi:10.3390/foods12213941)
Supplement: Supplementary file 1 [file foods-12-03941-s001.zip › foods-2646894-supplementary.pdf]

Supplementary document

Table S1. The ingredients of sample blank used for method validation.

Non-alcoholic beverage<br>(Traditional Chrysanthemum)	Yogurt<br>(Natural flavor)	Snack<br>(Spicy and sweet flavor squid-seafood snack)
- Chrysanthemum extract 94 %<br>- Rock sugar 5.95 %<br>- Gardenia Fruit 0.05%	- Milk 85%<br>- Non-fat milk powder 1.6%<br>- Concentration protein milk 0.09 %	- Squid 45%<br>- Surimi 40%<br>- Wheat flour 5%<br>- Tapioca starch 4 %<br>- Seasoning 3.5 %<br>- Sugar 2%<br>- Salt 0.5%

Table S2. Conversion factor for each steviol glycoside to calculate steviol equivalent [57].

**Steviol glycosides**	**Conversion factor**
Steviol	1.00
Stevioside	0.40
Rebaudioside A	0.33
Rebaudioside B	0.40
Rebaudioside C	0.33
Rebaudioside D	0.28
Rebaudioside F	0.34
Dulcoside A	0.40
Rubusoside	0.50
Steviolbioside	0.50

Table S3. Chemical profiling of selected steviol glycosides for mass spectrum references.

Derivatives of steviol glycosides	Empirical formula	M/z	RT (min.)
Rebaudioside D	$C_{50}H_{80}O_{28}$	1127.4710	2.9
Rebaudioside A	$C_{44}H_{70}O_{23}$	965.4223	6.8
Stevioside	$C_{38}H_{60}O_{18}$	803.3689	7.4
Rebaudioside F	$C_{38}H_{68}O_{22}$	935.4095	8.9
Rebaudioside C	$C_{44}K_{70}O_{22}$	949.4261	9.9
Dulcoside A	$C_{38}H_{60}O_{17}$	787.3725	10.4
Rubusoside	$C_{32}H_{50}O_{13}$	641.3154	13.6
Rebaudioside B	$C_{38}H_{60}O_{18}$	803.3669	14.5
Steviolbioside	$C_{32}H_{50}O_{13}$	641.3163	14.9
